# Unraveling the Global Phylodynamic and Phylogeographic Expansion of *Mycoplasma gallisepticum*: Understanding the Origin and Expansion of This Pathogen in Ecuador

**DOI:** 10.3390/pathogens9090674

**Published:** 2020-08-19

**Authors:** Laura De la Cruz, Maritza Barrera, Liliam Rios, Belkis Corona-González, Carlos A. Bulnes, Adrian A. Díaz-Sánchez, Jose A. Agüero, Evelyn Lobo-Rivero, Lester J. Pérez

**Affiliations:** 1 Facultad de Ciencias Veterinarias, Universidad Tecnica de Manabi, Manabi 13132, Ecuador; laudelacvel@hotmail.com (L.D.l.C.); maritza.barrevalle@gmail.com (M.B.); cbulnes60@gmail.com (C.A.B.); 2 Reiman Cancer Research Laboratory, Faculty of Medicine, University of New Brunswick, Saint John, NB E2L 4L5, Canada; Liliam.rios@gmail.com; 3 Risk Analysis of the Caribbean Region, OIE Collaborating Centre for Diagnosis, Centro Nacional de Sanidad Agropecuaria (CENSA), Mayabeque 32700, Cuba; bcorona@censa.edu.cu (B.C.-G.); jaaguero@censa.edu.cu (J.A.A.); elobo@censa.edu.cu (E.L.-R.); 4 Department of Biology, University of Saskatchewan, Saskatoon, SK S7N 5E2, Canada; adiasanz88@gmail.com; 5 OIE Reference Laboratory for Diagnostic of Mycoplasmas (MycoLab), Mayabeque 32700, Cuba; 6 Department of Clinical Veterinary Medicine, College of Veterinary Medicine, University of Illinois, Urbana, IL 61802, USA; 7 Veterinary Diagnostic Laboratory, College of Veterinary Medicine, University of Illinois, Urbana, IL 61802, USA

**Keywords:** *Mycoplasma gallisepticum*, *mgc2* gene, temporal diversification, phylogeographic approach, phylodynamic expansion

## Abstract

*Mycoplasma gallisepticum* (MG) is among the most significant problems in the poultry industry worldwide, representing a serious threat to international trade. Despite the fact that the *mgc2* gene has been widely used for diagnostic and molecular characterization purposes, there is a lack of evidence supporting the reliability of this gene as a marker for molecular epidemiology approaches. Therefore, the current study aimed to assess the accuracy of the *mgc2* gene for phylogenetic, phylodynamic, and phylogeographic evaluations. Furthermore, the global phylodynamic expansion of MG is described, and the origin and extension of the outbreak caused by MG in Ecuador were tracked and characterized. The results obtained strongly supported the use of the *mgc2* gene as a reliable phylogenetic marker and accurate estimator for the temporal and phylogeographic structure reconstruction of MG. The phylodynamic analysis denoted the failures in the current policies to control MG and highlighted the imperative need to implement more sensitive methodologies of diagnosis and more efficient vaccines. Framed in Ecuador, the present study provides the first piece of evidence of the circulation of virulent field MG strains in Ecuadorian commercial poultry. The findings derived from the current study provide novel and significant insights into the origin, diversification, and evolutionary process of MG globally.

## 1. Introduction

Infections with mycoplasmas represent a global threat for both public and animal health [1]. In poultry, *Mycoplasma gallisepticum* (MG) presents a broad range of clinical diseases, with chronic respiratory disease (CDR) in chickens [2] and infectious sinusitis in turkeys [3] as the most relevant manifestations. The global economic impact caused by these two diseases in terms of loss in meat and egg production, increase in embryo morbidity, as well as the burden associated with the control programs for MG, including antibiotic medication and vaccination [4], has been estimated to be around $780 million per year [5]. Hence, MG is among the most significant problems afflicting poultry worldwide and is considered a concern for international trade.

Taxonomically, mycoplasmas are classified within the phylum *Firmicutes*, class *Mollicutes*, order *Mycoplasmatales*, family *Mycoplasmataceae*. Thus, mycoplasmas are considered the self-replicating prokaryotes with the smallest genomes, characterized by the lack of a cell wall and containing the minimum set of organelles essential for growth and replication [6]. As a result of their limited genetic information, protein expression in mycoplasmas is restricted, lacking many enzymes and metabolic pathways, which makes them depend on their hosts [7] and compete with the host cells for metabolic substrates such as lipid precursors, purines, and pyrimidines [8].

Managing MG infected flocks is a difficult task that relies on the combined efforts of different control policies including medication, vaccination, and strict biosecurity measures [9]. Although antibiotic medication could be an effective tool to control the clinical signs related to MG infections, this strategy fails to eliminate infection from a flock. It is also relevant to consider that mycoplasmas are resistant to a wide spectrum of antibiotics. Since these microorganisms lack a cell wall, they are unaffected by β-lactamic antibiotics such as penicillin or cephalosporins. Mycoplasmas are also naturally resistant to rifampicin, and due to the absence in these microorganisms of lipopolysaccharides and folic acid synthesis, they are also resistant to polymyxins, sulfonamides, first-generation quinolones such as nalidixic acid, and trimethoprim [4]. Likewise, vaccination against MG has shown to be effective only to control clinical signs, especially in multi-age commercial egg production flocks [9]. An effective biosecurity program has been suggested as adequate enough to maintain flocks free of mycoplasma infections [10]. However, on one hand, this strategy relies on efficient and reliable monitoring systems enabling early detection during an outbreak, and on the other hand, there are large concentrations of poultry in small geographic areas. These aspects have negatively impacted most of the biosecurity programs, hindering the control of MG worldwide. 

Successful infection by mycoplasmas relies on their mechanisms of pathogenesis, adherence to the host cells being a critical step [11]. In the case of MG, this species also has the capacity to cross the mucosal barrier, which plays a major role in its systemic spreading [12]. This property has been linked to the gliding motility mechanism, providing MG with an adaptative advantage. A recent report by Indikova et al. (2014) identified the *mgc2* gene within the *mgc* locus involved in the motility of MG, highlighting the role of this gene as a key virulence factor of MG [13]. 

It is relevant to note that the *mgc2* gene has been widely employed for diagnostic purposes for the detection of MG using qPCR assays [14,15,16] and even the current technologies based on loop-mediated isothermal amplification (LAMP) [17]. Likewise, the *mgc2* gene has been extensively used for molecular characterization of MG isolates from different countries, including Jordan [18] and Egypt [19], as well as for phylogenetic approaches in isolates from the United Kingdom [20], Russia [21], and Iran [22]. However, despite the practical use of the *mgc2* gene to detect and characterize different field strains of MG, there is a lack of information regarding the reliability of the *mgc2* gene as a molecular marker for phylogenetic, phylodynamic, and phylogeographic approaches. Similarly, despite worldwide concern regarding the economic losses caused by MG, there is quite limited knowledge regarding the strains of MG circulating in Central and South America. Indeed, whereas the presence of antibodies against MG has been detected in backyard chickens in Argentina, Brazil, and Ecuador, with the exception of Brazil, there is no information regarding the circulation and characteristics of MG in commercial poultry flocks in these countries. In this regard, several outbreaks of respiratory diseases with clinical signs compatible with CDR in chicken flocks were presented in the province of Manabí in Ecuador during 2018 and 2019. Therefore, the present study mainly aimed to assess the reliability of the *mgc2* gene as a molecular marker for phylogenetic, phylodynamic, and phylogeographic approaches. Furthermore, using this molecular marker, the global phylodynamic expansion of MG as a relevant pathogen in poultry is described. Likewise, by using the *mgc2* gene as a phylogeographic marker, the origin and extension of the outbreak caused by MG in Ecuador were tracked. The findings derived from the current study provide novel and significant insights into the origin, diversification, and evolutionary process of MG globally. On the other hand, the characterization of the outbreak of MG in Ecuador using molecular and clinical approaches sheds light on additional aspects of the epidemiological situation of this pathogen in the South American region. 

## 2. Results and Discussion

Since the first documented characterization of chronic respiratory disease (CDR) in chickens in 1926 [23], countless efforts have been made to control and eradicate this infectious disease, starting from the identification of its causative agent [24] and the implementation of novel vaccines and control measures [25] to the sequencing of the whole genome of MG in this new “genomic era” [26,27,28]. In this regard, whole genome sequencing facilitates comparison between different strains, including field isolates and vaccines, enabling the identification of determinants of virulence [29]. However, the use of the complete genome in molecular epidemiology studies is unfeasible due to several aspects. For instance, MG’s complete genome length is around 1Mb; hence, its use in phylogenetic, phylodynamic, or phylogeographic approaches would be extremely costly computationally [30]. On the other hand, the whole genome of MG contains recombinant portions that affect the phylogenetic information [31], and sequencing the whole genome is difficult to accomplish by most diagnostic laboratories. Most significantly, since the complete genome contains all the genes, the action of the evolutionary forces on all of them could be different, with three putative scenarios [32]. Firstly, on highly conserved genes such as those encoding for polymerases or functional enzymes, the action of the purifying selection predominates, leading to deleterious or non-viable variants that disappear from the genetic spectrum, resulting in an underestimation of the mutation rates and consequently an increase in phylogenetic noise or loss of the phylogenetic signal between strains [33]. A second scenario involves the influence of the genes under neutral selection, identified like those under random changes (mutations or genetic drift) that can represent a better adaptation or a disadvantage to a future condition but do not necessarily respond to an environmental variation [34]. Therefore, establishing reliable relationships between different taxa based on this type of gene is basically impossible since the forces driving the genetic diversity are random genetic drifts that do not follow a directional selection [34]. Finally, those genes influenced by selective selection, which represents an adaptive advantage caused by previous pressure of the environment, are considered the best phylogenetic markers [32]. These types of genes contain information about population demographic processes and have the potential to measure shifts in population size arising from environmental changes and adaptation [35]. Consequently, the *mgc2* gene of MG encodes for a cytadhesin which is exposed in the surface of the microorganism membrane and is under continuous pressure imposed by the immune response from the infected hosts [36]. Considering all the aspects described above, the current study addressed as its main aim the theoretical and practical evaluation of the *mgc2* gene as a reliable marker to perform phylogenetic, phylodynamic, and phylogeographic inferences for MG globally.

**Figure 1:**
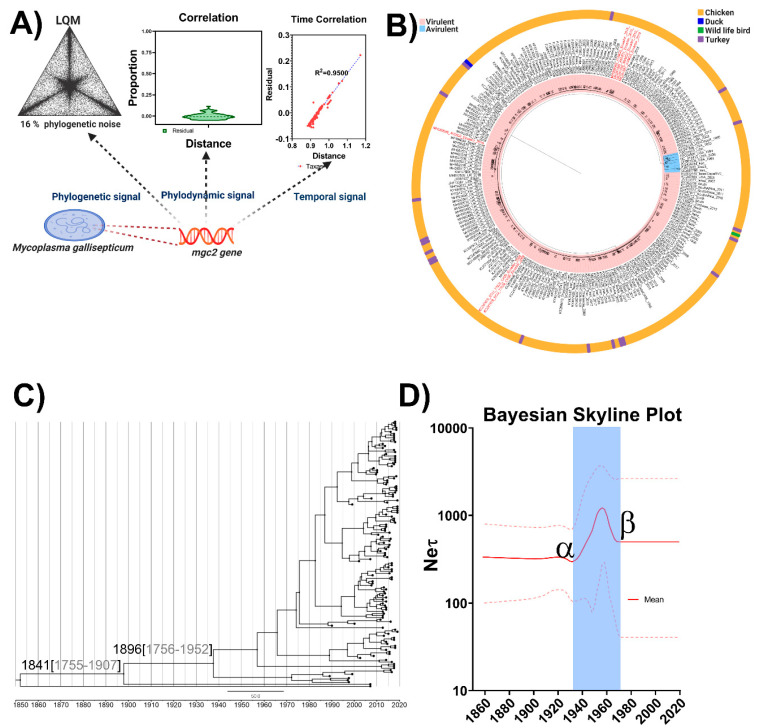
Phylogenetic, phylodynamic, and temporal assessment and application of *mgc2* gene of *Mycoplasma gallisepticum*. (**A**) Evaluation of phylogenetic noise (left), phylodynamic signal (center), and temporal signal (right) of the *mgc2* marker used. Left panel represents the likelihood mapping of *mgc2* sequences; the dots inside the triangles represent the posterior probabilities of the possible unrooted topologies for each quartet (LQM). Numbers indicate the percentage of dots in the center of the triangle corresponding to phylonetic noise. The center panel represents the node distribution based on the genetic distance and the proportion of residuals; violin plots were generated using the Graphpad Prism software 8.4.1 (1992–2020, Graphpad Prism Software Inc., San Diego, CA, USA). The right panel represents the regression of root-to-tip genetic divergence. Representation created with Biorender.com. (**B**) Phylogenetic tree based on *mgc2* sequences using all no-redundant genomes available at GenBank and maximum likelihood (ML) method; the GenBank IDs for all the sequences, with country and year of isolation, are shown. The main two lineages related to virulence are denoted by designations and colors (virulent: red, non-virulent: blue): the taxa names of the Ecuadorian MG strains are denoted in red, and the bootstrap values are denoted. In addition, to facilitate the host source information for each strain/isolate, a color code was included (chicken: dark yellow, duck: blue, wild bird: green, and turkey: purple). (**C**) Evolutionary history reconstruction of MG strains globally. Maximum clade credibility (MCC) tree based on *mgc2* sequences from all the field strains of MG, the estimation of the most probable year for the MRCA for the ancestor and the emergence of MG, as well as the 95% highest probability density (HPD) are denoted. (**D**) Bayesian skyline plot (BSP) using an exponential, uncorrelated clock model. The *x*-axis is in units of year, and the *y*-axis represents the logarithmic scale of Neτ (where Ne is the effective population size and τ is the generation time). The *x*-axis is in units of year, and the *y*-axis represents the logarithmic scale of Neτ (where Ne is the effective population size and τ is the generation time). The period from the development of the commercial poultry to the starting use of the antibiotics in poultry is highlighted by blue shadow.

Initially, the phylogenetic noise of the dataset was investigated through likelihood mapping. The percentage of dots falling in the central area of the triangles yielded 16% of unsolved topologies (Figure 1A). This result strongly supports the use of the region proposed as a phylogenetic marker since a value less than 30% for phylogenetic noise is accepted as a reliable value in phylogenetic inference [37]. As previously described by Rios et al. (2017), the evaluation of the strength of a dataset is a key factor to ensure a reliable phylogenetic design. Phylogenetic noise can mislead phylogenetic inference; hence, the identification of optimal levels of noise exclusion reduces the number of topologies that are not significantly worse than the optimal tree. This step allows for a more robust inference of phylogeny and stronger conclusions about the evolutionary character [38]. The phylodynamic signal was assessed by plotting the residual of node distribution based on the genetic distance. The violin plot (Figure 1A) shows that the topology obtained based on the *mgc2* gene is correctly recovered. This result was also supported by the fact that the *mgc2* gene solved a “stemmy tree” topology (see [39] for definition) with very short apical branches (see Supplementary Materials, Figure S1) and the evolutionary rate obtained from IQtree calculation to recovery the most basal split was 0.8542, which is the optimal rate for this type of topology [39]. This result highlights the accuracy of the marker assessed as the phylodynamic evaluator and therefore its ability to assess shifts in population size caused by environmental changes. On the other hand, it is well accepted that the use of an unreliable phylogenetic marker can strongly impact the utility of a set of characters (sequences) for inference of evolution [40]. In other words, determining the utility of a topological resolution to estimate the time scale of a given phylogenetic problem is another critical feature to consider. A recent algorithm implemented in the TempEst software enables the exploration of the degree and pattern of the temporal signal in the datasets [41]. This approach has been successfully applied to guarantee a closer relationship between genetic information and the temporal pattern [42,43]. In the current study, the data obtained from the *mgc2* marker showed a linear correlation between genetic distance and temporal structure, with an R^2^ = 0.95 (Figure 1A). Hence, it can be inferred that *the mgc2* marker is a reliable estimator for the temporal reconstruction of MG. 

The phylogenetic reconstruction based on ML methodology revealed a clear split between the virulent field strains of MG and those nonvirulent/vaccine strains with statistic support indicated by a bootstrap value of 89% (Figure 1B). In addition, several clades included in the virulent group of MG were segregated with statistic support based on bootstrap values >50%; however, since a clear relationship between clade-specific and phenotype-specific could not be established, an additional classification based on clades was found to be worthless (Figure 1B). The topological reconstruction from the *mgc2* obtained in the current study concurred with the overall reconstruction obtained in previous publications using the *mgc2* marker to phylogenetically classify different strains of MG [20,21,22]. Notoriously, different research groups have suggested different strategies, targeting multiple genes in order to obtain a higher level of discrimination, including random amplified polymorphic DNA (RAPD) [44], gene-targeted sequencing (GTS) [45], and the most recent multilocus sequence typing assay (MLST) [46]. However, even though these could be useful strategies to provide a higher genetic resolution to classify and characterize MG, these approaches have several intrinsic issues. For instance, RAPD has shown problems with reproducibility, hampering inter-laboratory comparisons and long-term epidemiological analysis (reviewed in [44]). On the other hand, GST analysis of the four genes used (*mgc2*, *gapA*, MG*A_0319*, *IGSR*) showed a higher level of discrimination by *mgc2* than the remaining three [44]; therefore, the utility of these remaining three genes is arguable. Similarly, the promising methodology recently reported as the MLST tool [46] showed higher discriminatory power than the GST; however, it is relevant to note that this discriminatory power was based on the capacity of the tool to discriminate between different strains with different geographical locations [46]. Additionally, two major issues with this approach must be highlighted. Firstly, an association index that would allow a correlation between MLST genotype classification and geographic location was not conducted in the mentioned study [46]; therefore, the “discriminatory power” based on geographic location is a biased estimation, misleading the interpretation of the characterization of the MG strains. In addition, and more relevant, is the fact that the MLST tool lacked a temporal pattern of association between MLST genotypes, as was evidenced in Complex 2, where nine different MG strains were classified in the same node but isolated in a 13 year time range (from 1995 to 2008) (see Figure 1 in [46]). Hence, whereas the multitarget strategies mentioned could have a certain degree of utility in the genetic classification of MG, they lack the accuracy to be applied in molecular epidemiology studies.

From the path sampling (PS) and stepping-stone (SS) sampling methodologies, it was revealed that an exponentially growing population size model with an uncorrelated exponential clock was the best-fitted model for the phylogenetic marker *mgc2* and the dataset used (Supplementary Table S2). In this point, it is necessary to denote the need for a proper estimation of the Bayesian parameter-rich models for sequence evolution analyses. Whereas at least three consequential studies [47,48,49] have proven a higher level of reliability in the PS and SS methodologies over the Bayesian factor (BF), the harmonic mean estimator (HME), and a posterior simulation-based analog of Akaike’s information criterion through Markov chain Monte Carlo (AICM) [47], different research groups continue the evaluation of the molecular evolution based on a random selection of the evolutionary model or by using unreliable estimators [50,51], which leads to errors in the estimations of evolutionary rates, phylodynamic patterns, and tMRCA. Using the selected model, a Bayesian phylogenetic tree obtained for the global MG strains was characterized by a clear temporal structure (Figure 1C and Supplementary Materials Figure S1); the oldest samples tended to fall closer to the root of the tree, while the most recent samples were located at the most distal tips (for better visualization in Figure 1C, the tree was condensed but the pattern can be fully visualized in Supplementary Materials Figure S2). The mean of the tMRCA for the *mgc2* gene of MG was located at approximately the year 1841 (95% HPD from 1755 to 1907), with diversification and genetic establishment (emergence) located approximately in 1896 (95% HPD from 1756 to 1952) (Figure 1C). These results perfectly align with the historical description of MG outbreaks in poultry. The first description of a disease linked to MG was historically recognized in the report by Prof. Sydney Dodd from the Royal Veterinary College of London in 1905 [52]. In his report, Prof. Dodd explained that a previous report by Prof. M’Fadyean in 1893 described a very similar disease in turkeys that was found in October 1892. From the description of the case, four points were denoted by Prof. Dodd as distinctive between both cases, concluding that the two reports were describing different microorganisms [52]. However, unraveling these four points based on the current knowledge about MG, it can be concluded that, certainly, the first outbreak related to MG was described in the report by Prof. M’Fadyean in 1893 [53], since it is well known that MG is a motile mycoplasma [13] that causes adhesive pericarditis and it is not related with enteritis [54]. The closeness among the estimated values (Bayesian estimation) and the historical record about the emergence of MG are indicative of the accuracy and reliability of the methodology used and also suggest that the emergence of MG as a relevant pathogen in poultry is linked to the evolutionary events in the *mgc2* gene. This finding gains relevance if it is considered that mycoplasmas have special genome plasticity, with events of gene losses (which are commonly observed in symbiont genome evolution) but with extensive gene gain, a rare event in obligate pathogens with small genomes [55]. Indeed, in other species, i.e., *Mycoplasma agalatiae*, it has been proposed that 18% of the genome was acquired by horizontal gene transfer (HGT) from the Mycoides cluster [56]. Therefore, the *mgc2* gene was very likely acquired in MG by an HGT event through which MG could have acquired gliding motility as a functional advantage, facilitating the colonization of the hosts (turkey and chicken) and its consequent spread and expansion. After a brief search using the BLAST webtool, a potential parental that could have contributed as a donor was identified: *Mycoplasma imitans* (*MI*) has an *adhesine* gene with 60% identity with *mgc2*. *MI* is commonly isolated in concomitant infection with MG and it has been isolated from a broader range of hosts including ducks, geese, chicken, turkeys [57], and partridge [58]. However, to confirm this hypothesis, further analyses will be required.

The demographic inference of MG using the Bayesian skyline plot (BSP) is shown in Figure 1D. BSP essentially plots Neτ as a function of time. Neτ can be considered as a measure of relative genetic diversity that reflects the number of effective infections established by the microorganism [59]. The BSP revealed for MG maintenance in the Neτ from its emergence to the late 1920s (Figure 1D), with a sudden increase from 1926 to 1956, reaching the maximum level of genetic diversity between 1950 and 1956. From there, it suddenly decreased until 1970, followed by a constant Neτ value, suggesting stability in the diversity of the MG population for the last period (Figure 1D). The stability in the effective population size (Neτ) for MG during its first period of circulation reflects the establishment of an endemic situation in the regions where it was presented. However, the major factor that could have contributed to the sudden increase in the Neτ seems to be the development of the poultry per se. It is well documented that, during the late 1800s and early 1900s, most of the farmers had small flocks with ~ 200 birds; however, in 1923, with the invention of the first heated incubator, industrial chicken production started [60]. This event was followed by the massive effort supported by 55 nations from 1946 to 1948 to find better breeds for two separate purposes: maximum egg production and meat consumption [61]. By 1949, the global expansion of the selected breeds started and lasted until late 1959, when the commercial chicken industry was consolidated around the world. Hence, with this increase in the number of birds, an increase in animals infected with MG is highly probable, influencing the genetic diversity of the *mgc2* gene. The sudden decrease observed in Neτ from 1957 to 1970 could be linked to the use of antibiotics in poultry, which was approved by the mid-1950s (reviewed in [62]), mainly the use of tetracycline, which is effective against MG [4]. The change found in the trend from a decreasing Neτ value towards the maintenance of or constant Neτ could be associated with different aspects, including the emergence of strains resistant to the antibiotics in use, failure to establish proper control measures, and the banning of antibiotics in poultry in the 1970s [62], among others. However, two additional aspects deserve critical discussion. Firstly, the first vaccine against MG was applied in 1977 [63]; thereafter, at least two more live attenuated vaccines were developed and applied in poultry during the 1980s and 1990s (reviewed in [64]), but from our BSP analysis, it can be inferred that the application of all these vaccines did not impact the genetic diversity of MG, suggesting that vaccination against MG only helped to control the clinical signs linked with the disease and probably the productive indicators but did not control the infectivity of the agent. Therefore, it can be considered an inefficient tool (at least in its status quo) in the eradication of MG. Moreover, the fact that the mean Neτ maintained a higher value than the initial value is indicative that the global policies designed to eradicate MG have been unsuccessfully implemented.

The BaTS analysis revealed that the global trait association tests for phylogeographic structure rejected the null hypothesis of no association between geographic location and phylogeny at all spatial levels assessed for the MG strains included in the present study (Table 1 and Supplementary Materials Table S1). Therefore, from this result, it can be concluded that the sequences of the *mgc2* marker have a phylogeographic structure. The use of index ratios (IR) of the observed values to those expected under panmixis (where 0 indicates complete population subdivision and 1 suggests random mixing (panmixis)) allows the strength of the association between geography and phylogeny. Hence, the AI of 0.44 (0.38–0.53 CI 95%) suggests that the diversification and evolution of MG based on the *mgc2* marker is not homogeneous but rather presents a geographic structure. Indeed, analysis of MC statistics proved that the population subdivision for MG was significant for most of the localities, although samples from Ecuador, Australia, Thailand, India, Pakistan, Saudi Arabia, Colombia, Panama, Jordan, Venezuela, and Zimbabwe showed evidence of gene flow (Table 1). This feature could be a direct consequence of the poultry industry in these listed countries (except India), which depends on the genetic processes of overseas breeding companies. Meanwhile, Indian breeding involves a mix of imported breeds from the United States (US), Netherlands, United Kingdom (UK), and Israel [65]. Therefore, the panmixis found in all the listed countries can be linked to a constant gene flow exchange caused by regular importations for breeding purposes. The phylogeographic reconstruction approach was able to identify a single location for the root of the tree based on the global dataset of *mgc2* sequences for MG, with a posterior probability for state sp = 0.556 for the locality of the US (Supplementary Materials Figure S3). 

**Figure 2:**
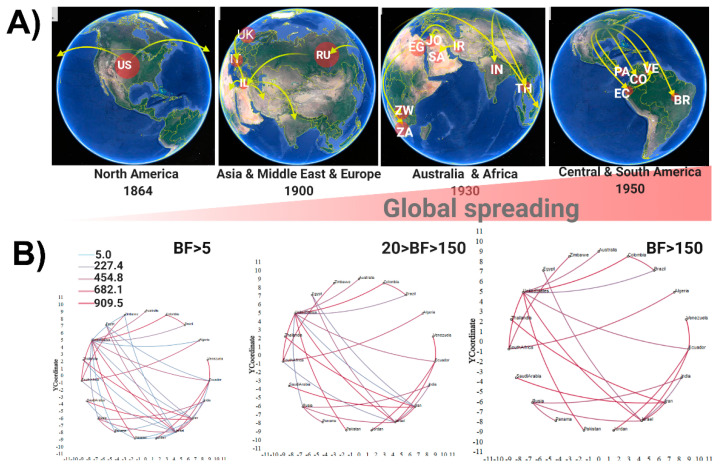
Global spatiotemporal dispersal of *Mycoplasma gallisepticum*. (**A**) Dispersal patterns inferred using discrete phylogeographic analysis of the *mgc2* dataset are shown for four time slices; the map was reconstructed using Google Earth from the SpreaD3 software output. (**B**) Most probable route for the transmission of MG values of BF are denoted, the connection map was directly taken from out file of the spreaD3 software. Country code was used based on two letters representation following *ISO3166*. Representation created with Biorender.com.

Applying globe animations using maps, the historical spreading of MG revealed the ancestor in the US in ~1864 (Figure 2A); the microorganism then propagated and spread outwards in the early 1900s, arriving in several locations in Europe, Asia, and the Middle East (Figure 2A). The close relationship among the US, UK, and Italy in the development of the modern poultry industry (with several crosses between different breeds from these three countries) can easily explain the spread of MG among these countries [61], whereas the links with Russia and Israel are not so clear. However, looking into the development of the poultry sector in Russia after the establishment of the “All-Russian Society of Poultry Farming” in 1896, there was an increased interest in establishing a commercial breed pedigree and several foreigner breeds were introduced through St. Petersburg, Moscow, and the Baltic provinces, which were mixed with national breeds to improve their resistance to winter (reviewed in [66]). Among the approximately 13 imported breeds, the American breeds *Brahams* and *Wyandotte* played a relevant role. By 1898, the Russian poultry had grown considerably in egg and meat production as well as exportation (see Table 1 in [66]), but the introduction of the American breeds seems to be the main source of the introduction of MG in Russia. 

In the case of Israel, it is necessary to clarify that, in 1900, there was not a State of Israel; the first Zionists settlement in Palestine occurred in 1896, followed by a second wave of immigration in 1904 (mainly from Russia), with the consequent establishment of the first “kibbutz” in 1909 based exclusively on Jewish Labor (reviewed in https://aub.edu.lb.libguides.com/c.php?g=342715&p=2477016). Thus, since the settlers carried with them all the media and animals to establish agricultural and farm production, these events could have caused the introduction of MG to this geographic area. MG arrived then in Africa and Australia by 1930 (Figure 2A), spreading through different means. On one hand, it is relevant that the development of and increased interest in Australian poultry started in 1950; therefore, most poultry production was in the hands of backyard producers and larger family operations. However, despite the lack of surveillance methods as early as 1940, Hart [67] described an outbreak of infectious sinusitis in turkeys as the first documented evidence of the circulation of MG. After this initial report, and for several years, a rise in the incidence of respiratory conditions affecting Australian poultry was noted, characterized by a trend of starting during the mid-summer months and reaching a peak in January, which were later confirmed as cases caused by MG [68]. Hence, our prediction that MG could have arrived in Australia during 1930 has a high degree of accuracy. On the other hand, the first hints about problems linked to MG in South Africa and Zimbabwe are described by Armour et al. [44] and Moretti et al. [69]; hence, due to the absence of reports of outbreaks caused by MG in the past in these two countries, and the lack of information about the development of poultry, it is difficult to establish a link between our phylogeographic approach and historical records. From the phylogeographic analysis, it was revealed that, by 1950, MG had been already propagated across the globe (Figure 2A), with the US as the main source of entry, reaching South America (including Panama, Ecuador, Colombia, Venezuela, and Brazil). Several questions could arise from this last result, mainly regarding why South America, having a strong economic nexus with the US, seems to be the last geographic area colonized by MG. Notoriously, before 1950, the local poultry was sustained by backyard producers that used local breeds such the Araucana chicken, among others [70], instead of imported breeds. It is important to highlight that the South American chickens had good economic indicators and were well adapted to the region since their introduction seems to be pre-Columbian [71]. With the advent of the modern poultry industry, several commercial flocks were installed in South America using the cosmopolitan commercial lineages [70], this being the most probable cause of introduction of MG into the region. Analyzing the support for the routes of transmission of MG using Bayes factor (BF) BF > 5 (Figure 2B), a high level of connection among different countries was revealed, evidencing the high degree of spreading of MG and its pandemic characteristic. The role of the US as a relevant route for the dissemination of this microorganism across the world was also evidenced by a BF > 150 (Figure 2B). This result reflects the fact that, despite the policies adopted to control MG, mainly in the US, these measures have been inefficient. 

**Figure 3:**
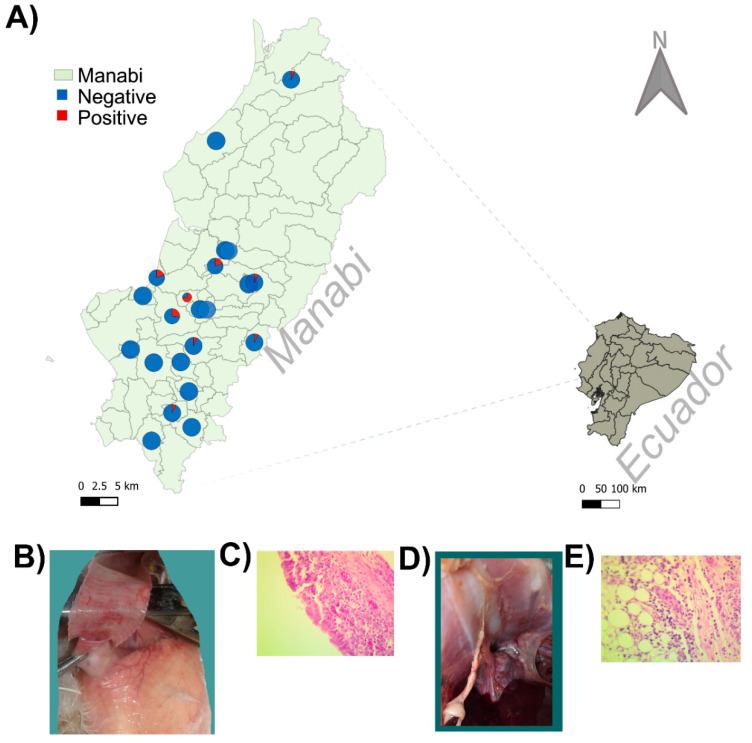
Distribution and pathological characterization of the *Mycoplasma gallisepticum* outbreak in Ecuador. (**A**) Ecuador map showing the geographic distribution of samples collected. Pie chart representing proportion of samples negative and positive are shown. Map was built using QGIS 3.10 (https://www.qgis.org.) (**B**–**E**) Representative pathologic lesions in the selected chicken: (**B**,**D**) gross observations; (**B**) tracheitis; (**D**) airsacculitis; (**C**,**E**) histopathological analysis; (**C**) trachea; (**E**) air sacs.

Within the global context of the emergence and expansion of MG, the current study also aimed to track the origin and to characterize the first outbreak of MG in commercial flocks in Ecuador. From the previous analysis, the first introduction of MG in Ecuador was estimated during the 1950s, concurring with the establishment and expansion of the Ecuadorian poultry industry, initiated with the importation of the BB breed chicks for hatcheries from the US [72]. Although the introduction of MG in Ecuador took place around 70 years ago, this pathogen was not considered a serious problem in Ecuadorian poultry until a few years ago. This characteristic could be associated with the fact that the Ecuadorian poultry industry lacks a breeding process, importing chicks for the hatchery directly from the US, which could have helped to mitigate the extensive propagation of MG in the country as well as the lack of significant outbreaks. The Ecuadorian MG isolates were located in three different clades (Figure 1B), suggesting different introductions to the country without genetic links between them. Despite the current study focusing only on the geographic location of Manabí, the results obtained suggested a broad circulation of MG across the province (Figure 3A). From a total of 22 flocks analyzed, nine tested positive for MG (40.9%) by conventional PCR (Figure 3A). The within-flock prevalence of broilers ranged between 6.7% and 40% (Figure 3A). The fact that the samples were obtained from chickens presenting clinical signs contrasts with the low number of positive samples yielded by the PCR, mainly considering the MG-positive samples vs. total number of swabs tested (24 out of 330 samples). This result could be influenced by several aspects including the improper preservation of the clinical material, degradation of target, among others discussed by Perez et al. [73], but previous studies have also shown for other respiratory pathogens a significant gap in sensitivity between based-on-gel PCR and real-time PCR (qPCR) techniques [74]. Hence, the use of a based-on-gel PCR could have led to fewer positive samples, emphasizing the urgent need for the implementation of a qPCR methodology to be used in the diagnosis and surveillance of MG in the poultry industry in Ecuador. The post-mortem examination of selected animals exhibited the presence of pathological lesions compatible with MG infection, including tracheitis characterized by the presence of a discrete catarrhal to fibrinous exudate with congestion and dispersed hemorrhagic foci in the tracheal mucosa, mainly located in the upper third of this organ (Figure 3B), and acute catarrhal airsacculitis which was characterized by thickening in the air sacs with opacity and presence of focal fibrinous exudate (Figure 3D). The pathological lesions found were further confirmed by hematoxylin/eosin stains on histological sections (Figure 3C,E). Thus, in the trachea, it was observed the presence of discrete hyperplasia of the goblet cells and infiltration of mononuclear cells in the mucosa, mixed with some heterophiles, as well as congestion and edema (Figure 3C). In the air sacs, moderate infiltration of lymphocytes mixed with plasma cells, macrophages, and formation of structures like lymphoid nodules was visualized (Figure 3E). In addition, fibrinous exudate displaying discrete infiltrate, with a predominance of heterophiles and moderate vascular changes, was also observed. In all cases, the presence of MG was confirmed by PCR. Thus, all the pathological and histopathological lesions found correspond with previous descriptions of infections by MG caused by virulent field strains [54]. Although a previous report warned about the circulation of MG by detecting antibodies in a total of 100 animals in backyard poultry [75], the current study represents the first molecular and pathological characterization of an outbreak caused by MG in commercial poultry in Ecuador. 

## 3. Materials and Methods

### 3.1. Sequence Dataset and Multiple Alignment

A sequence dataset containing all of the *mgc2* gene sequences available in the GenBank database (http://www.ncbi.nlm.nih.gov/) was downloaded. From the 499 sequences obtained, only unique sequences were included in the study. Sequences were filtered using DAMBE software buffer [76,77] and the sequence obtained from the most antique isolate/strain was kept as original (Supplementary Materials Table S1). From this sequence dataset, additional information was subtracted, such as year of isolation and geographical location and host (Supplementary Materials Table S1). This dataset also included the sequences obtained in the current study from Ecuadorian isolates (Supplementary Materials Table S1, denoted in bold case). Sequences were aligned using the algorithm ClustalW method included in the program BioEdit Sequence Alignment Editor [78].

### 3.2. Evaluation of mgc2 as Phylogenetic, Phylodynamic, and Phylogeographic Marker 

#### 3.2.1. Likelihood Mapping

The phylogenetic signal of the sequence dataset was investigated by likelihood mapping, as described by Rios et al. [59]. Briefly, 100,000 random quartets generated using TreePuzzle were analyzed. In this strategy, if more than 30% of the dots fall in the center of the triangle, the data are considered unreliable for phylogenetic inference purposes [79].

#### 3.2.2. Phylodynamic Noise Evaluation

To determine the temporal structure of the dataset, a regression of root-to-tip genetic distance was performed using TempEst software [41]. In addition, the phylodynamic signal was assessed by node distribution based on the genetic distance and the proportion of residuals generated by TempEst software [41].

#### 3.2.3. Phylogeographic–Trait Association 

The association between phylogeny and the pattern of the geographical distribution of MG based on the *mgc2* gene was assessed as described by Alfonso et al. [80]. Briefly, the phylogeographic resolution of the *mgc2* gene was assessed using the software BaTS [81]. In this approach, the trait of geographic region for the sequence dataset was assessed by the association between the different phylogenetic relationships of the MG strains used and the geographical locations (countries). Values of the association index (AI), parsimony score (PS) statistics, and the level of clustering in individual locations using the monophyletic clade (MC) size statistic were calculated based on the posterior samples of trees produced by MrBayes 3.2.7 [82] using the BaTS program. The null distribution for each statistic was estimated with 1000 replicates of state randomization.

### 3.3. Phylogenetic Analysis, Evolutionary History, Phylodynamic, and Phylogeographic Analyses 

To estimate the phylogenetic relationships between the different MG strains, the dataset was analyzed as described [30]. Briefly, since no recombinant sequences were identified from all the sequences included in the dataset (Supplementary Materials Table S1), a maximum likelihood (ML) phylogenetic tree was computed using the IQ-TREE program (Nguyen, Schmidt, von Haeseler, & Minh, 2015). IQ-TREE was also used to select the best-fitted model from the analyzed dataset. Confidence levels for the branches were determined by the Shimodaira test and 1000 replicates of bootstrap in IQ-TREE. 

To estimate the time of the most recent common ancestor (tMRCA), we followed the methodology described by Rios et al. [59]. Briefly, initially, a Bayesian model selection was performed by estimating model marginal log-likelihood through the path sampling (PS) and stepping-stone (SS) sampling methods described by Baele and Lemey [48], using coalescent demographic models including parametric models, nonparametric models, and different molecular clocks (Supplementary Materials Table S1). In addition, a Bayesian skyline plot (BSP) for MG strains to infer the population dynamics in terms of changing levels of relative genetic diversity (Neτ) through time was performed. For BSP analysis, data were collected and plotted using Graphpad Prism software 8.3 (1992–2019, Graphpad Prism Software LLC., San Diego, CA, USA). In all cases, the Markov chain Monte Carlo (MCMC) chains were run for 2.5 × 10^8^ generations to obtain an ESS > 250, and the first 10% of trees were discarded as “burn-in”. Finally, trees were visualized and edited in FigTree.

The spatial diffusion dynamics of MG was estimated following the methodology described in Barrera et al. [83]. Briefly, a Bayesian discrete phylogeographic approach was conducted by using a standard continuous-time Markov chain (CTMC) model with Bayesian stochastic search variable selection (BSSVS) to model the geographic transmission of MG from the selected sequence of the dataset (Supplementary Materials Table S1, sequences with year of isolation available). The resulting maximum clade credibility (MCC) phylogenetic tree obtained by TreeAnnotator was summarized, and Bayes factor (BF) support for individual transitions between discrete locations was computed using Spread D3 v0.9.681. We interpreted the strength of statistical support as described by Fusaro et al. [84]. Briefly, we determined positive support for 5 < BF < 20, strong support for 20 < BF < 150 and very strong support for BF > 150.

### 3.4. Ecuadorian Outbreak Tracking and Description 

#### 3.4.1. Ethics Statement 

All experimental protocols were approved by the Bioethics Committee of Universidad Técnica de Manabí, protocol #R01003018, following guidelines for the conduct of animal experiments and international standards for animal welfare included in the regulations for the animal sampling of Article 134 of the Animal Welfare Law included in the National Constitution of the Republic of Ecuador. All methods were conducted in accordance with relevant guidelines and regulations.

#### 3.4.2. Sample Collection 

A total of 330 tracheal swabs were collected from 22 commercial raising broiler flocks located in the province of Manabí, Ecuador. The sampling was carried out during 2018 and 2019, selecting 15 chickens/flock with ages between five and six weeks old which were showing clinical signs compatible with MG infection. Tracheal swab sampling was conducted following the procedures described by Poveda [85]; also, each sample was individually kept in 1mL phosphate buffer saline 1X (1X PBS). Moreover, in each flock, from the total number of animals sampled, five chickens were sacrificed and submitted for histopathological studies.

#### 3.4.3. Nucleic Acid Isolation, MG Detection, and Sequencing 

DNA from the tracheal swab samples was isolated following the heat-shock method described by Hernandez et al. [86]. For the diagnosis, the PCR based on gel targeting the *mgc2* gene, as reported by Lysnyansky et al. [87], was performed. Subsequently, all the positive samples were submitted for amplification using the same primer pair described by Lysnyansky et al. [87] but using Platinum Taq High Fidelity DNA Polymerase (Thermo Fisher Scientific, Waltham, MA, USA), according to the manufacturer’s instructions. All the amplification products obtained were purified from the agarose gel using QIAquick Gel Extraction Kit (Qiagen GmbH, QIAGEN, Hilden, Germany) and submitted for sequencing (bidirectional), which was conducted under BigDyeTM terminator cycling conditions by an external laboratory (Macrogen Korea, Beotkkot-ro, Geumcheon-gu, Seoul, Korea). The quality of each sequence obtained was visually analyzed using ChromasPro version 1.5 (http://www.technelysium.com.au/chromas.html) and assembled using BioEdit version 5.0.9 [78]. Sequence similarity was checked against sequences deposited in the EMBL/GenBank using a BLAST search on the NCBI site (http://www.ncbi.nlm.nih.gov). Nucleotide sequences obtained for the *mgc2* gene were submitted to the GenBank database under the accession numbers (MH089585, MH089586, MK241885-MK241887).

#### 3.4.4. Histopathology Analysis

All the tissue samples collected were fixed in 10% formalin for 24 h, dehydrated, and embedded in wax. The embedded samples were then cut into sections (5 μm) and stained with hematoxylin and eosin stain [88]. Histopathological lesions of air sacs and trachea were assessed and examined under a light microscope, as described by Sprygin et al. [21].

## 4. Conclusions

The current study provides novel and significant insights into the emergence and global expansion of MG as a significant pathogen affecting the poultry industry. The results obtained support the use of the virulent gene *mgc2* as a reliable phylogenetic, phylodynamic, and phylogeographic marker useful in studies of molecular epidemiology for MG locally and globally. As a relevant aspect of the phylodynamic analysis, the current study denoted the failures in the current policies of control of MG, highlighting the urgent need to implement improved strategies that include more sensitive methodologies of diagnosis and more efficient vaccines that eliminate the microorganism, not only solving the problems of the clinical signs and the productive indicators. 

Framed in Ecuador, the present work is the first study that provides evidence that virulent MG field strains are circulating in Ecuadorian commercial poultry. The phylodynamic and phylogeographic analyses suggested that these strains were introduced to Ecuador by the importation that is conducted from the US. Likewise, this study highlights the urgent need to establish a program of control and surveillance in Ecuador to tackle the circulation and expansion of MG in the country.

## Figures and Tables

**Table 1:** Phylogeny–trait association tests of the phylogeographic structure of *mgc2* gene for *Mycoplasma gallisepticum* using BaTS.

Statistic	IR (CI 95%)	Observed Mean	Expected Mean	Significance
AI	**0.44 (0.38–0.50)**	**6.6 (5.37–7.82)**	**14.92 (15.58–14.16)**	**<0.001**
PS	**0.57 (0.56–0.58)**	**70 (67.0–74.0)**	**123.79 (127.26–119.80)**	**<0.001**
MC (EC)	ND	1.27 (1.0–2.0)	1.03 (1.0–1.12)	1.0
MC (IL)	ND	**2.58 (1.0–3.0)**	**1.10 (1.0–1.49)**	**<0.001**
MC (AU)	ND	1.20 (1.0–2.0)	1.02 (1.0–1.07)	1
MC (TH)	ND	1.04 (1.0–1.0)	1.02 (1.0–1.07)	1
MC (US)	ND	**2.87 (2.0–3.0)**	**1.60 (1.17–2.13)**	**0.007**
MC (UK)	ND	**2.1 (1.0–4.0)**	**1.70 (1.10–2.30)**	**<0.001**
MC (ZA)	ND	**2.70 (1.0–6.0)**	**1.13 (1.0–1.65)**	**0.011**
MC (EG)	ND	**4.03 (2.0–7.0)**	**1.60 (1.17–2.19)**	**0.001**
MC (IR)	ND	**1.60 (1.0–2.0)**	**1.06 (1.0–1.26)**	**0.001**
MC (BR)	ND	**2.63 (2.0–3.0)**	**1.10 (1.0–1.41)**	**0.001**
MC (IN)	ND	1.00 (1.00–1.00)	1.00 (1.00–1.00)	1
MC (ZW)	ND	**1.66 (1.0–2.0)**	**1.06 (1.0–1.28)**	**0.002**
MC (IT)	ND	**2.54 (1.0–4.0)**	**1.06 (1.0–1.28)**	**0.005**
MC(PK)	ND	1.00 (1.00–1.00)	1.00 (1.00–1.00)	1
MC (SA)	ND	1.00 (1.00–1.00)	1.00 (1.00–1.00)	1
MC (CO)	ND	1.00 (1.00–1.00)	1.00 (1.00–1.00)	1
MC (PA)	ND	1.00 (1.00–1.00)	1.00 (1.00–1.00)	1
MC (JO)	ND	1.00 (1.00–1.00)	1.00 (1.00–1.00)	1
MC (VE)	ND	1.00 (1.00–1.00)	1.00 (1.00–1.00)	1
MC (RU)	ND	**6.94 (4.0–11.0)**	**1.66 (1.22–2.24)**	**0.001**
MC (DZ)	ND	1.00 (1.00–1.00)	1.00 (1.00–1.00)	1

**Note:** Significant values are denoted in bold case. Country abbreviation was followed by the two-letter country code according to ISO3166.

## Supplementary Materials

The following are available online at https://www.mdpi.com/2076-0817/9/9/674/s1, Table S1. Sequences used in the study; Table S2. Bayesian model selection based on path sampling and stepping-stone algorithm. Figure S1. Phylogenetic tree for the *mgc2* gene of *Mycoplasma gallisepticum*. Topological organization of the phylogenetic tree obtained from the dataset. The support values of the node are shown; the “stemmy” organization of the tree concurs with the evolutionary rate obtained and the residual plot vs. genetic. The main two lineages related to virulence are denoted by designations and colors (virulent: red, non-virulent: blue). Figure S2. Maximum credibility clade tree from the BEAST software package. The temporal structure is visualized and represented for the whole topology in a green triangle and for specific nodes in red triangles. Figure S3. Phylogeographic reconstruction of the global dataset tree for the *mgc2* gene of *Mycoplasma gallisepticum.* Posterior probabilities for state are shown.
