# Supplementary material for: Unraveling the Global Phylodynamic and Phylogeographic Expansion of Mycoplasma gallisepticum: Understanding the Origin and Expansion of This Pathogen in Ecuador
Source: Pathogens. 2020 Aug 19;9(9):674. doi: 10.3390/pathogens9090674 (PMC7557814; doi:10.3390/pathogens9090674)
Supplement: Supplementary file 1 [file pathogens-09-00674-s001.pdf]

TableS1. Sequences used in the study

No.	GenBank ID	Country	Year of isolation	Host
1	AY556291	Israel	2000	Chicken
2	AY556298	Israel	2000	Chicken
3	AY556302	Australia	1997	Chicken
4	KX268616	Thailand	2015	Chicken
5	AY556297	Israel	1999	Chicken
6	KX268631	Thailand	2015	Chicken
7	NC_018413	USA	2008	Chicken
8	KY362210	South Africa	2006	Chicken
9	KY362216	South Africa	2013	Chicken
10	KY362205	South Africa	2009	Chicken
11	KY362204	South Africa	2007	Chicken
12	KY362209	South Africa	2006	Chicken
13	KY362212	South Africa	2003	Chicken
14	KY362206	South Africa	2005	Chicken
15	KU577604	Australia	1985	Chicken
16	KY421064	Egypt	NA	Chicken
17	JQ770175	Australia	NA	Chicken
18	JQ770177	USA	NA	Chicken
19	MH102389	Egypt	2018	Chicken
20	GQ436784	Iran	NA	Chicken
21	KJ019176	Brazil	2013	Chicken
22	KP279741	India	NA	Chicken
23	KP279743	India	NA	Chicken
24	KJ019177	Brazil	2013	Chicken
25	KJ019173	Brazil	2011	Chicken
26	KP261894	India	NA	Chicken
27	GQ436786	Iran	NA	Chicken
28	GQ422751	Egypt	NA	Chicken
29	KJ019172	Brazil	2011	Chicken
30	KJ019175	Brazil	2012	Chicken
31	KP300754	India	NA	Chicken
32	KJ019171	Brazil	NA	Chicken
33	GQ436785	Iran	NA	Chicken
34	KJ019174	Brazil	2011	Chicken
35	AY556234	USA	1974	Chicken
36	AY556235	USA	1975	Chicken

37	AY556236	USA	1975	Chicken
38	AY556238	USA	1984	Chicken
39	AY556230	USA	1958	Chicken
40	AY556246	USA	1996	Chicken
41	AE015450	USA	2003	Chicken
42	CP006916	USA	2011	Chicken
43	CP001873	USA	NA	Chicken
44	CP003506	USA	1994	Chicken
45	CP003508	USA	1994	Chicken
46	CP003512	USA	2001	Chicken
47	AY556253	USA	1996	Chicken
48	KC130907	Zimbabwe	2012	Chicken
49	AY556228	USA	1980	Chicken
50	KC247870	South Africa	2011	Chicken
51	KC247872	South Africa	2011	Chicken
52	KC247871	South Africa	2010	Chicken
53	AY556301	Australia	1996	Chicken
54	KX268619	Thailand	2015	Chicken
55	KY491037	Iran	2013	Chicken
56	KM107807	Italia	NA	Turkey
57	KM107805	Italia	NA	Game bird
58	KM107808	Italia	NA	Turkey
59	KF874278	Pakistan	2013	Chicken
60	KF874279	Pakistan	2013	Chicken
61	KF874281	Pakistan	2013	Chicken
62	KF874280	Pakistan	2013	Chicken
63	KF874283	Pakistan	2013	Chicken
64	JX981943	Egypt	NA	Chicken
65	JX981945	Egypt	NA	Chicken
66	HQ591358	Egypt	NA	Turkey
67	EF462343	USA	1994	Chicken
68	HQ591357	Egypt	NA	Turkey
69	KJ364633	Egypt	2013	Duck
70	KP691072	Egypt	2014	Chicken
71	KT992784	Egypt	NA	Chicken
72	MF773877	Egypt	2017	Chicken
73	KP135564	Egypt	2014	Chicken
74	KY404987	Egypt	2016	Chicken
75	MG428412	Iran	2017	Chicken
76	MG428413	Iran	2017	Chicken
77	FJ234839	Egypt	NA	Chicken
78	HQ591355	Egypt	NA	Chicken

79	HQ591359	Egypt	NA	Chicken
80	KY012362	Iran	2016	Chicken
81	HQ591356	Egypt	NA	Chicken
82	MG676446	Egypt	2017	Chicken
83	MG820791	Egypt	NA	Chicken
84	MG820792	Egypt	NA	Chicken
85	KY467403	India	2015	Chicken
86	KX650154	Iran	2015	Turkey
87	KX650156	Iran	2016	Turkey
88	KY421187	Iran	2016	Chicken
89	KY421188	Iran	2016	Chicken
90	KY467400	India	2015	Chicken
91	KY651219	Iran	2016	Turkey
92	KY651221	Iran	2016	Chicken
93	KY651225	Iran	2016	Turkey
94	KY651226	Iran	2016	Turkey
95	KY651227	Iran	2016	Turkey
96	KY491030	Iran	2015	Chicken
97	KY491031	Iran	2012	Chicken
98	KY491032	Iran	2016	Chicken
99	KY491033	Iran	2015	Chicken
100	KY651220	Iran	2016	Turkey
101	KY491038	Iran	2015	Chicken
102	KY491039	Iran	2015	Chicken
103	KY467401	India	2015	Chicken
104	KY467402	India	2105	Chicken
105	KM107803	Italia	NA	Turkey
106	FJ395202	Pakistan	NA	Chicken
107	KT943467	Egypt	NA	Chicken
108	EU939449	Israel	NA	Chicken
109	JX981932	Egypt	NA	Chicken
110	JX981931	Egypt	NA	Chicken
111	JN113342	Israel	1997	Chicken
112	JN113382	Israel	2009	Chicken
113	JN113343	Israel	1978	Chicken
114	MG149560	Saudi Arabia	2016	Chicken
115	JX981938	Egypt	NA	Chicken
116	KC247897	USA	1978	Chicken
117	HQ143377	Israel	1995	Chicken
118	KC247873	Colombia	2008	Chicken
119	GQ859596	Egypt	2009	Chicken
120	KC247878	Panama	2008	Chicken

121	HQ143372	Egypt	2008	Chicken
122	KC247886	USA	2010	Chicken
123	HQ143374	Egypt	2009	Chicken
124	KC247890	Jordan	2009	Chicken
125	KC247893	Thailand	2008	Chicken
126	KC247895	USA	1995	Chicken
127	KC247896	USA	1960	Chicken
128	KC247880	Venezuela	2008	Chicken
129	MG676447	Egypt	2017	Chicken
130	FJ972632	Russia	NA	Chicken
131	MF773876	Egypt	2017	Chicken
132	MG149559	Saudi Arabia	2015	Chicken
133	KY126378	Pakistan	2015	Chicken
134	KP892754	Algeria	2012	Turkey
135	JX981927	Egypt	NA	Chicken
136	JX981926	Egypt	NA	Chicken
137	MF496039	Egypt	2016	Chicken
138	KM107804	Italia	NA	Chicken
139	JN113377	Israel	2007	Chicken
140	KM107806	Italia	NA	Chicken
141	FJ972630	Russia	2005	Chicken
142	FJ965776	Russia	2005	Chicken
143	FJ965777	Russia	2008	Chicken
144	FJ965779	Russia	2008	Chicken
145	FJ965780	Russia	2008	Chicken
146	FJ965781	Russia	2007	Chicken
147	FJ965782	Russia	2008	Chicken
148	FJ965783	Russia	2005	Chicken
149	FJ965785	Russia	2007	Chicken
150	FJ965786	Russia	2005	Chicken
151	FJ965790	Russia	2005	Chicken
152	FJ965792	Russia	2006	Chicken
153	FJ965793	Russia	2006	Chicken
154	FJ965795	Russia	2007	Chicken
155	FJ965796	Russia	2008	Chicken
156	FJ965797	Russia	2007	Chicken
157	FJ965798	Russia	2007	Turkey
158	FJ965799	Russia	2006	Chicken
159	FJ965800	Russia	2005	Chicken
160	FJ965801	Russia	2005	Chicken
161	KC247883	USA	2008	Chicken
162	FJ965791	Russia	2005	Chicken

163	FJ965778	Russia	2008	Chicken
164	EU315110	Egypt	NA	Chicken
165	JN113387	USA	2010	Chicken
166	KC247881	Venezuela	2008	Chicken
167	KC247892	India	2005	Chicken
168	FJ965787	Russia	2008	Chicken
169	MH089585	Ecuador	2018	Chicken
170	MK241885	Ecuador	2019	Chicken
171	MK241886	Ecuador	2019	Chicken
172	MK241887	Ecuador	2019	Chicken
173	MH089586	Ecuador	2019	Chicken
174	KC247875	Ecuador	2009	Chicken
175	KC247876	Ecuador	2009	Chicken
176	MH592485	UK	2015	Chicken
177	MH592486	UK	2016	Turkey
178	MH592492	UK	2016	Chicken
179	MH592494	UK	2016	Chicken
180	MH592497	UK	2016	Chicken
181	MH592488	UK	2016	Turkey
182	MH592493	UK	2016	Chicken
183	MH592489	UK	2016	Chicken
184	MH592501	UK	2016	Chicken
185	MH592502	UK	2016	Chicken
186	MH592503	UK	2016	Turkey
187	MH592507	UK	2016	Chicken
188	MH592510	UK	2016	Chicken
189	MH592515	UK	2016	Chicken
190	MH592517	UK	2016	Chicken
191	MH592518	UK	2016	Chicken
192	MH592519	UK	2016	Chicken
193	MH592521	UK	2016	Chicken
194	MH592522	UK	2016	Chicken
195	MH592525	UK	2016	Chicken
196	MH592527	UK	2016	Chicken
197	MH592532	UK	2016	Chicken
198	MH592535	UK	2016	Chicken
199	MH592506	UK	2016	Chicken
200	MH592539	UK	2016	Chicken
201	MH592526	UK	2016	Chicken
202	MH592542	UK	2016	Chicken
203	MH592504	UK	2016	Turkey
204	MH592509	UK	2016	Chicken

205	MH592505	UK	2016	Turkey
206	MH592511	UK	2016	Chicken
207	MH592533	UK	2016	Chicken
208	MH592520	UK	2016	Chicken
209	MH592524	UK	2016	Chicken
210	MH592537	UK	2016	Chicken
211	MH592523	UK	2016	Chicken
212	MH592534	UK	2016	Chicken
213	MH592512	UK	2016	Chicken
214	MH592514	UK	2016	Chicken
215	MH592516	UK	2016	Chicken
216	MH592528	UK	2016	Chicken
217	MH592529	UK	2016	Chicken
218	MH592530	UK	2016	Chicken
219	MH592531	UK	2016	Chicken

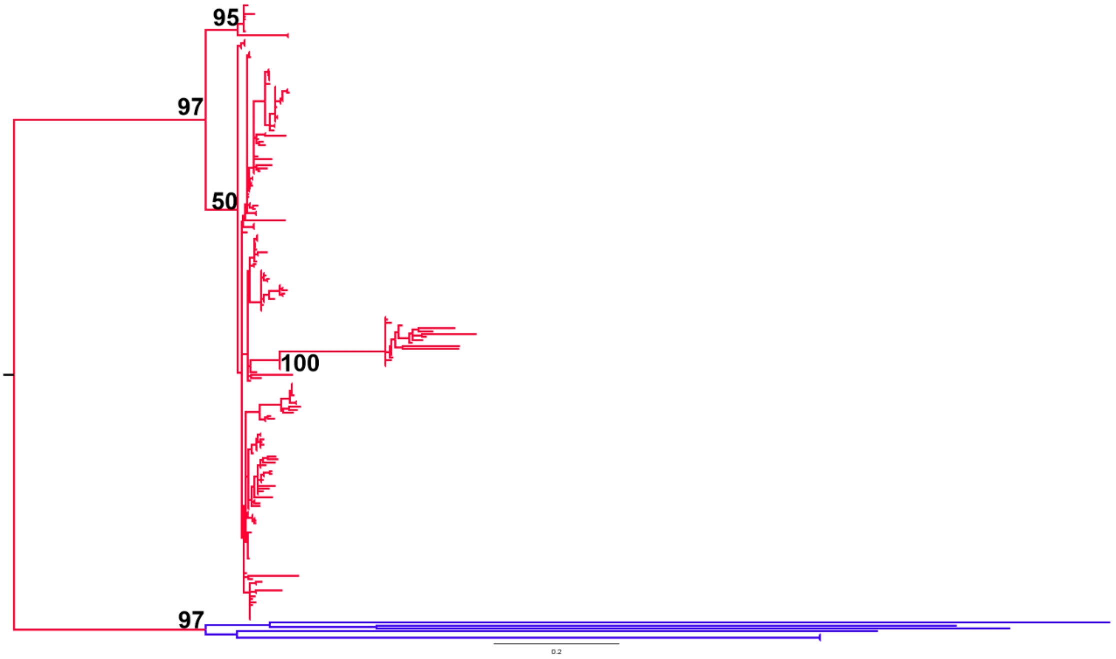

FigureS1. Phylogenetic tree for the *mgc2* gene of *Mycoplasma gallisepticum*. Topological organization of the phylogenetic tree obtained from the dataset. The support values of the node are shown, the "stemmy" organization of the tree concurs with the evolutionary rate obtained and the residual plot vs genetic. The main two lineages related to virulence are denoted by designations and colors (virulent: red, non-virulent: blue)".

TableS2. Bayesian model selection based on path sampling and stepping stone algorithm.

Clock Model	Coalescent	Path Sampling (PS)	Stepping Stone (SS)
SC	Const	-4950.432489	-4951.477534
	Exp	-4942.069671	-4942.918444

	BSP	-4879.9	-4881.499089
URL	Const	-4841.220551	-4843.172802
	Exp	-4831.8	-4832.357086
	BSP	-4842.251261	-4842.957649
RLC	Const	-4875.037934	-4876.406125
	Exp	-4869.323606	-4871.626471
	BSP	-4876.141073	-4878.588321
FLC	Const	-4890.25631	-4891.40946
	Exp	-4893.03855	-4893.367141
	BSP	-4881.618569	-4882.616919

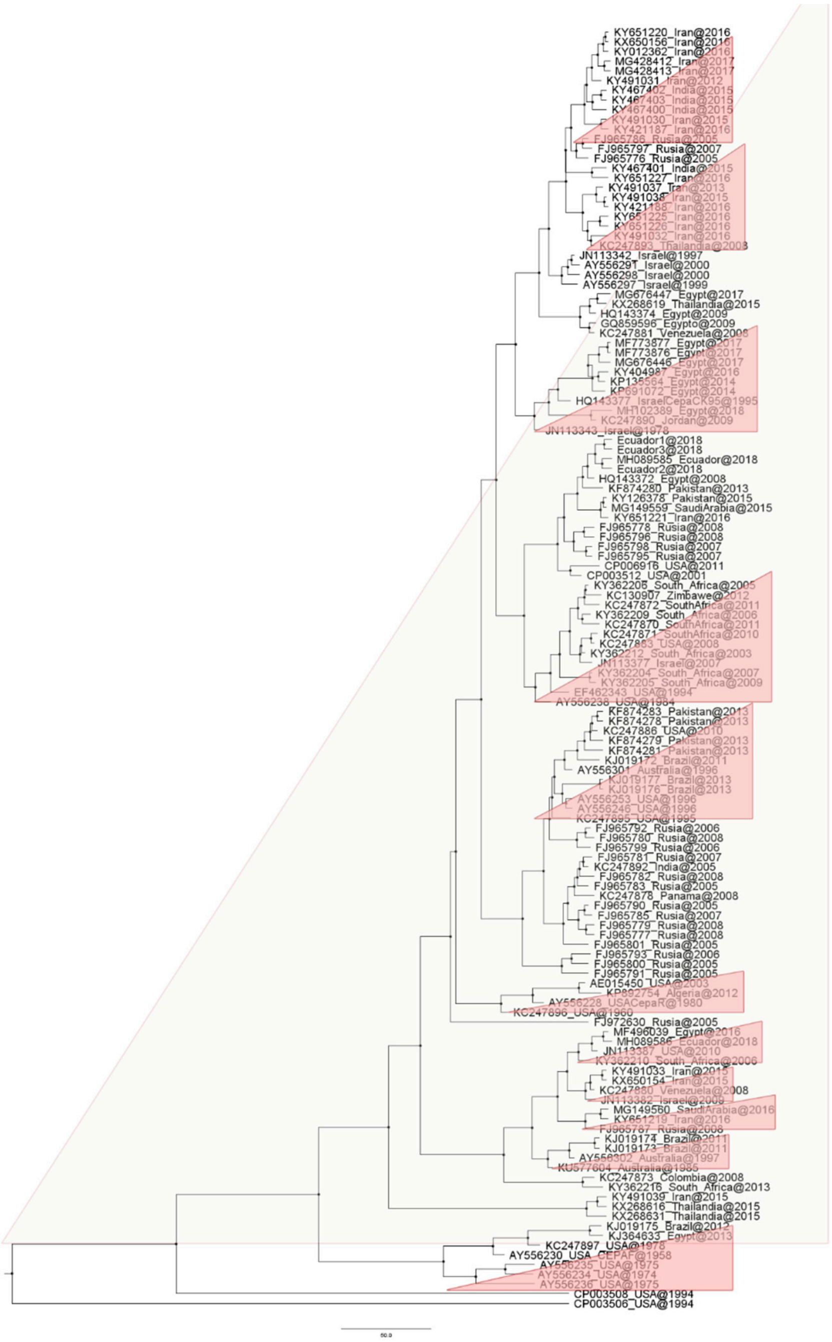

FigureS2. Maximum credibility clade tree from the BEAST software package. The temporal structure is visualized and represented for the whole topology in a green triangle and for specific nodes in red triangles.

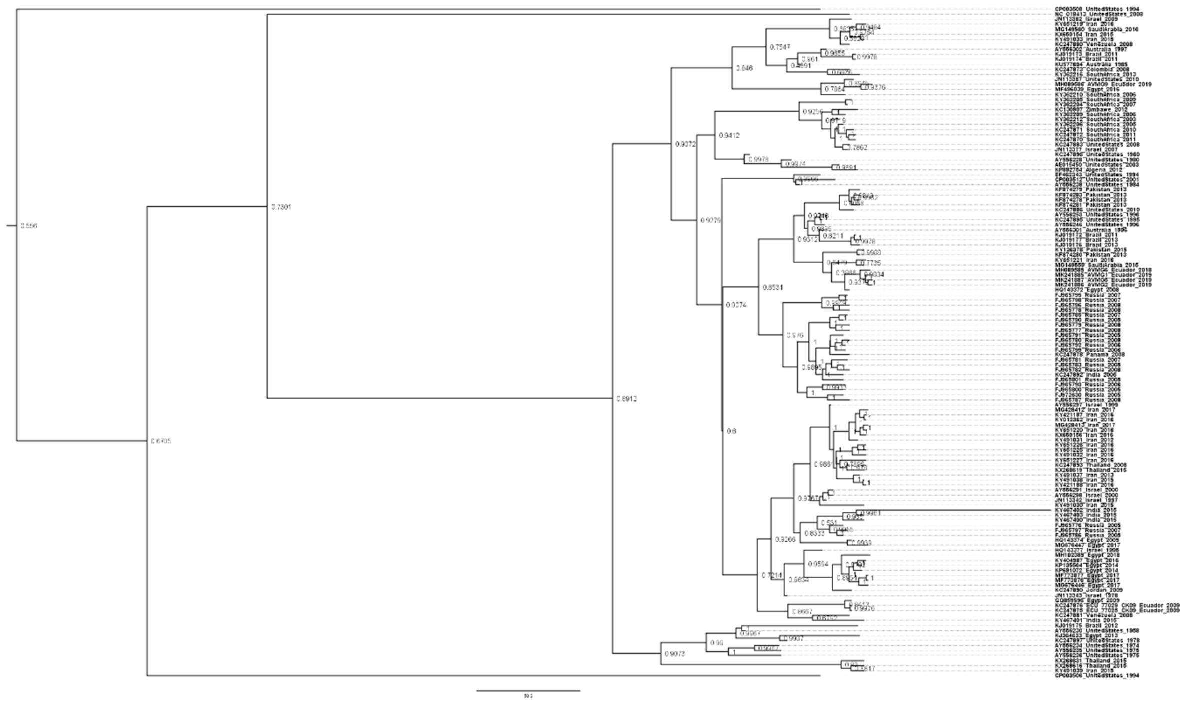

FigureS3. Phylogeographic reconstruction of the global dataset tree for the *mgc2* gene of *Mycoplasma gallisepticum*. Posterior probabilities for state are shown.
